# Development of a Robust, Simple, and Affordable Human Gait Analysis System Using Bottom-Up Pose Estimation With a Smartphone Camera

**DOI:** 10.3389/fphys.2021.784865

**Published:** 2022-01-05

**Authors:** Aditya Viswakumar, Venkateswaran Rajagopalan, Tathagata Ray, Pranitha Gottipati, Chandu Parimi

**Affiliations:** ^1^Department of Electrical and Electronics Engineering, Birla Institute of Technology and Science Pilani, Hyderabad, India; ^2^Department of Computer Science and Information Systems, Birla Institute of Technology and Science Pilani, Hyderabad, India; ^3^Independent Researcher, Cary, NC, United States; ^4^Department of Civil Engineering, Birla Institute of Technology and Science Pilani, Hyderabad, India

**Keywords:** OpenPose, kinematics, markerless system, gait, smartphone

## Abstract

Gait analysis is used in many fields such as Medical Diagnostics, Osteopathic medicine, Comparative and Sports-related biomechanics, etc. The most commonly used system for capturing gait is the advanced video camera-based passive marker system such as VICON. However, such systems are expensive, and reflective markers on subjects can be intrusive and time-consuming. Moreover, the setup of markers for certain rehabilitation patients, such as people with stroke or spinal cord injuries, could be difficult. Recently, some markerless systems were introduced to overcome the challenges of marker-based systems. However, current markerless systems have low accuracy and pose other challenges in gait analysis with people in long clothing, hiding the gait kinematics. The present work attempts to make an affordable, easy-to-use, accurate gait analysis system while addressing all the mentioned issues. The system in this study uses images from a video taken with a smartphone camera (800 × 600 pixels at an average rate of 30 frames per second). The system uses OpenPose, a 2D real-time multi-person keypoint detection technique. The system learns to associate body parts with individuals in the image using Convolutional Neural Networks (CNNs). This bottom-up system achieves high accuracy and real-time performance, regardless of the number of people in the image. The proposed system is called the “OpenPose based Markerless Gait Analysis System” (OMGait). Ankle, knee, and hip flexion/extension angle values were measured using OMGait in 16 healthy volunteers under different lighting and clothing conditions. The measured kinematic values were compared with a standard video camera based normative dataset and data from a markerless MS Kinect system. The mean absolute error value of the joint angles from the proposed system was less than 9^0^ for different lighting conditions and less than 11^0^ for different clothing conditions compared to the normative dataset. The proposed system is adequate in measuring the kinematic values of the ankle, knee, and hip. It also performs better than the markerless systems like MS Kinect that fail to measure the kinematics of ankle, knee, and hip joints under dark and bright light conditions and in subjects with long robe clothing.

## Introduction

Human gait refers to the pattern of walking in human beings. A gait cycle comprises of a series of body movements leading to locomotion in humans. The duration between a heel strike and the next heel strike of the same foot is called one gait cycle. Gait analysis is an objective and quantitative approach to measure/characterize walking. Predominately gait analysis comprises of kinematics (joint angle), kinetics (forces), and electromyography (muscle activity) (Perry and Burnfield, 2010). Gait analysis is used in fields such as medicine, sports-related biomechanics, ergonomics, etc.

Developments in the field of gait analysis systems for measuring kinematics go way back to the era of Aristotle, but significant contributions came from Willhelm and Weber in the seventeenth century (Baker, 2007). They were successful in marking the position of limbs for distinct phases of the gait cycle. The invention of photographic cameras and films in the early nineteenth century changed the field of gait analysis as finer details of human motion could be recorded and studied. Muybridge had pioneered the chronophotographic study of human motion analysis (Baker, 2007). He invented the zoopraxiscope, a device that could play back the captured phases of a gait cycle. The next breakthrough in gait analysis came with the advent of powerful digital computers and image processing techniques. The first video processing system for human gait analysis was developed by Davis et al. (1991) in the year 1991. He used passive reflective markers and image processing algorithms to track important joints in the human body. A significant limitation of reflective markers is that their accuracy is very sensitive to lighting conditions (Clayton, 1996). Also, these fiducial markers (i.e., reflective markers), when placed on the body are susceptible to choppy movements resulting from the sliding of skin over the bones (Reinschmidt et al., 1997). This introduces noise in the measured gait parameters. In recent years passive reflective marker-based motion capture systems (Colyer et al., 2018) are being widely used. These marker systems (such as VICON) have become popular in gait analysis since the gold standard approaches (Gasparutto et al., 2017), like measuring kinematics using intra-cortical bone pins, are invasive. However, these marker systems are expensive, requiring elaborate laboratory settings and skilled personnel to calibrate and collect data.

Also, placing reflective markers on subjects’ bodies could be considerably time-consuming (Ceseracciu et al., 2014). This can be a constraint in the case of stroke and spinal cord injury patients. Also, the data collection cannot be done in a clinical setting, and the subjects have to be transported to a laboratory facility. This is a significant issue in developing countries (Kumar et al., 2018), such as India (Patil et al., 2017), where resources are limited. Other methods to measure gait kinematics include using inertial sensors, goniometers, and accelerometers (Muro-De-La-Herran et al., 2014). Compared to the marker system, these sensors can be placed on the body in lesser time. However, similar to passive markers the sensors are attached using tapes. These attachments are subject to displacement during movement. Also, they have to be placed in locations where intrusion to the most sensitive regions in the patient’s body is required. Access to these places is very hard in cases of certain clothing (non-western) especially in patients with disabilities. These sensors are connected between the patient and the data acquisition system using long wires that could cause noise and drop out in signals. With the development of microcontrollers and integrated circuits, it became possible to manufacture wearable devices for measuring gait metrics (Tao et al., 2012). However, these wearable devices can cause physical discomfort and affect the normal walking of the subject. This is especially true if the devices are heavy and are connected by cables to some power source. Even though wireless sensors are available, these systems are considerably expensive. They also require adequate calibration and a controlled environment along with trained professionals to operate.

Recent markerless gait analysis techniques try to overcome the above drawbacks of marker and sensor systems by using depth imaging such as Microsoft Kinect (Gabel et al., 2012). Kinect system based approaches make use of coded infrared grids to develop a 3D map of real world objects. But these studies have shown errors in skeletal tracking due to the depth imaging algorithm failures in the presence of occlusions and non-distinguishing depths (Han et al., 2013). Moreover, Kinect’s skeletal tracking fails under bright ambient lighting (El-Laithy et al., 2012; Livingston et al., 2012). Further, our study (Parimi et al., 2017) using the Kinect system demonstrated that knee joint angles calculated using Kinect had appreciable accuracy only when the knees were visible, i.e., when not covered by clothing. Even though the Kinect system is affordable and less intrusive, the accuracy is poor under various lighting and clothing conditions.

Markerless systems try to estimate the pose by two approaches; (a) Generative and (b) Discriminative (Colyer et al., 2018). In the generative approach, a skeletal model is initially considered and iteratively refined to match the pose in the image data obtained. In the discriminative approach, the pose estimation is obtained from the overall image data itself. Even though the discriminative approach avoids iterative model fitting compared to the generative approach, it suffers from the creation of exemplary data (Colyer et al., 2018).

Clinical gait analysis is becoming an integral part of patient rehabilitation and is routine in many centers for patient management (Whittle, 1996). It is also widely used in other outdoor sports and clinical biomechanics applications.

Existing methods for gait data capturing are either expensive or intrusive. This study attempts to design an affordable, non-intrusive smartphone camera-based system that has adequate accuracy for the given applications. Such a system is needed for small clinics, especially in developing countries. It should also be noted that robe-like garments (sarees, thwabs, burkha, dhotis, etc.) are typical in many countries, and capturing the gait parameters in such cases can be challenging. This study also aims to address this issue. Smartphone usage around the world is very high. Even in developing nations, smartphone usage is increasing irrespective of socio-economic status. These smartphones have cameras with high resolutions. Using these cameras for gait analysis could be affordable and accessible even in economically backward regions of the world. If the analysis of smartphone camera videos can give robust gait parameters, the system can be considered “easy-to-use.” Recent developments in computer vision algorithms help estimate the pose of a person with adequate accuracy from videos. Such an algorithm can help develop a system that will not need intrusive markers or cables placed on the patient. When clothing covers the joints (long robe-like dresses) of the human body, conventional gait analysis systems fail. Low lighting can also hinder the capture of accurate joint angles. Our experience with existing gait analysis systems such as VICON, goniometers/accelerometers/Kinect has shown that they are not robust under these conditions. Further, no study has been performed to evaluate the effectiveness of using a smartphone video-based system for measuring kinematics under these conditions. In these systems, the user is compelled to wear specific clothing that is not always desirable. Depth imaging-based systems such as Kinect also fail to perform in different clothing conditions and extreme lighting conditions. OpenPose algorithm overcomes the above drawback with the help of a well-trained CNN followed by the novel approach of part affinity fields (PAF) which considers both the position and orientation of the joints in space. It should be noted that the computational expense of the method is significantly less than most of the gait analysis methods since OpenPose reduces the data to 2D pose estimation. The primary objective of this study is to assess the efficacy of the OpenPose based gait analysis system (OMGait) under various ambient lighting and clothing conditions. OMGait is also a non-intrusive and affordable system that can be easily deployed in developing countries.

## Methodology

### Details of the Gait Analysis System

**FIGURE 1:**
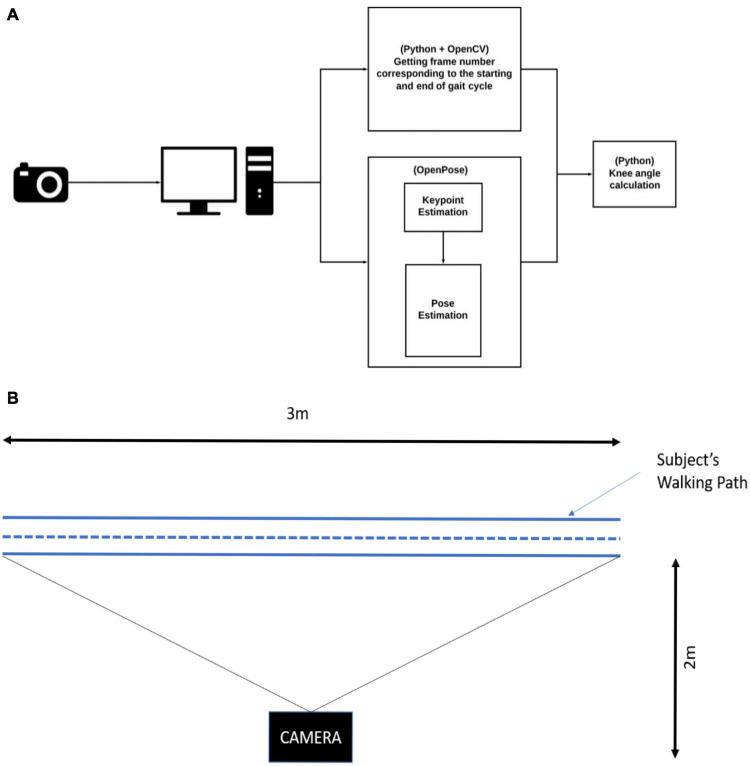
**(A)** Schematic of the complete proposed simple system to measure kinematics of the gait, **(B)** location of the camera with respect to the subject walkway.

Considering the above aspects, a system (Figure 1) was developed using a generic smartphone (XIAOMI REDMI GO F1) with 800 × 600 pixel images at an average rate of 30 frames per second and the pose estimation algorithm OpenPose (Cao et al., 2018). The authors note that this is a starting point in developing a more robust and accurate gait kinematics system.

The video capturing system consists of a smartphone mounted on top of a tripod stand at a height of 75 cm and placed parallel to the subject’s walkway (Figure 1B). Subjects walked in a straight line, parallel to a video capturing system. The camera is placed at such a distance to capture 3 m of the walkway in its field of view. Video data was downloaded from the phone onto a computer through a wireless network. The videos were processed using OpenPose.

Pose estimation methods employ either a top-down or bottom-up approach to generate human pose (Ning et al., 2019). The top-down approach comprises of the following steps, (i) Estimation of the pose (ii) Extraction of the key-points from the estimated pose. The precision of the extracted keypoints is dependent on the estimated pose. Hence, the top-down method is prone to errors in cases where capturing the keypoints is difficult such as photos with crowds and those taken in low-light conditions. Moreover, this approach is computationally expensive for multi-person pose estimation as each pose is estimated independent of each other. Hence, the bottom-up approach (Li et al., 2018) is used in this study as an alternative to the top-down approach. It involves two steps, (i) estimation of keypoints, and (ii) establishing a relation between the keypoints to generate the pose. Unlike the top-down approach, key point detection is the first step and is independent of any sort of pose estimation. Therefore, keypoint and pose estimation using the bottom-up approach is much faster and less prone to error when compared to the top-down approach. OpenPose is a 2D multi-person pose estimation library based on the bottom-up approach. Using OpenPose 135 vital body points can be detected in the absence of fiducial markers. We used OpenPose to extract anatomical joint coordinates in the lower extremities.

OpenPose employs a CNN for both key point detection and association. The key points are detected with a confidence score, a measure of the accuracy of detected key points. Keypoint association is estimated using PAF (Cao et al., 2018). PAFs are two-dimensional vector fields that encode the position and orientation of the limbs (or pairs). A bipartite graph is created using the estimated keypoints. The weights of this bipartite graph are calculated using the line integral of the PAFs estimated for each pair. An assignment algorithm is then applied to determine the part candidates that fit the pairs. Subsequently, merging is done to detect the complete skeleton. The design paradigm of Open Pose facilitates high precision human joints estimation from digital images/videos, which is used to develop a human gait analysis system (OMGait).

**FIGURE 2:**
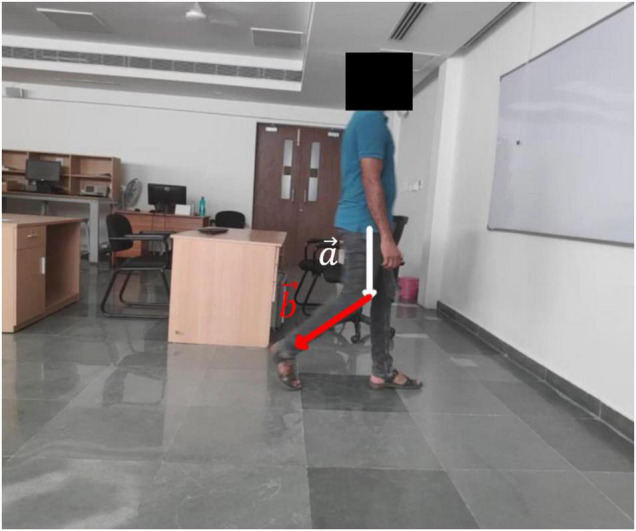
Shows how knee angle is calculated once the key points are located. The knee angle is calculated using the vector dot product. From the hip, knee, ankle coordinates obtained from the skeletal data two vectors are constructed. The first vector begins at the hip and ends at the knee while the second one begins at the knee and ends at the ankle. The knee angle (θ) is given by the following equation: $\theta =\cos^{-1}\frac{\vec{a}.\vec{b}}{\left|a\right|\,\left|b\right|}$.

Open Pose has been trained to produce three distinct pose models. Out of the three models, BODY_25, which uses 25 points, is used for this study. It can be seen that BODY_25 is the most exhaustive pose estimation model (Cao et al., 2018). Each frame of the downloaded video was processed using OpenPose to generate BODY_25 skeletal data. Walking trials on which the OpenPose failed to identify the joint locations correctly were discarded from further data processing. From the generated skeletal data, two dimensional coordinates of knee, hip and ankle joints along with the tips of the feet were extracted for each of the legs. From these coordinates, the required kinematic angles (hip, knee and ankle flexion/extension) were calculated. For example, the knee flexion/extension angle calculation is shown in Figure 2.

### Validation of the System

#### Collection of Data

Figure 1 shows the experimental setup. A total of 16 (12 male and 4 female) healthy student volunteers participated in this study. All the participants were explained the experimental protocol and usage of data. All of them gave verbal consent before participation. The mean ± standard deviation of the weight, height, and age of the participants were 70.3 ± 12.3 kg, 170.5 ± 8.69 cm, and 26 ± 3.5 years, respectively. The participants initially dressed in regular pants.

OMGait should work in uncontrolled environments namely, lighting and clothing. To study the effect of ambient lighting on the kinematic measurements, the subjects were asked to walk under the following lighting conditions; (1) Dim light with doors and window shields at an average radiance of 50 lux (2) Normal lighting condition with an average radiance of 320 lux and (3) Bright lighting at an average radiance of 9,800 lux.

Different clothing conditions were included in our study. Conventional clothing (e.g., shorts or tight trousers) is where most of the lower extremity joints are visible to the gait capturing system, and non-conventional clothing is where the limbs cannot be differentiated by the gait capturing system (robe like dresses such as kilt, thawb, dhoti, saree).

Kinematic measurements were also carried out in uncontrolled environments such as having multiple people in the background.

#### Data Analysis

Knee, hip, and ankle flexion extensions are calculated using the keypoints recognized using OpenPose. Subjects were allowed to start and end the gait cycles as per their convenience over a 3 m walkway. From each walking trial, three complete gait cycles were obtained per subject. Traditionally the start of a gait cycle is marked by either a heal strike or a push-off. In our case, the video capturing system is not synchronized with the phases of the gait cycle. Hence, we define the gait cycle as the time between two peaks of the joint angles. The average gait cycle for a subject is obtained by finding the mean of all these gait cycles. This is repeated for every joint angle. To obtain the gait analysis data for a given set of subjects in a particular scenario (lighting, clothing, etc.), the mean of the average gait cycles of all the subjects corresponding to that scenario is used.

The reproducibility of the data was assessed by comparing the mean and standard deviation of different observations on the same subject recorded over different sessions. The feasibility of the proposed system was assessed by comparing the average ankle, knee, and hip angle values from all 16 subjects with the mean and standard deviation values from the normative database. We observed that the measured kinematic angles were out of phase with the normative angles. We synchronize for knee angle phase shift by horizontally translating it till the global maxima of the measured and normative angle fall on the same vertical line. For the hip and ankle angles, we do the same but with the global minima. Let m(t) and n(t) be the measured and normative gait angles at an instance “t.” The horizontal shift “h_min_” applied to knee angles is that shift “h” which minimizes the difference between maximum of m(t) and n(t).


$${\text{h}}_{\text{min}}={\text{argmin}}_{\text{h}}\left[\max\{\text{m}\left(\text{t-h}\right)\}\text{—max}\left\{\text{n}\left(\text{t}\right)\right\}\right]$$

For the hip and ankle angles the minimum of the measured and the normative data are matched using the horizontal shift as given below:


$${\text{h}}_{\text{min}}={\text{argmin}}_{\text{h}}\left[\mathrm{ma}\text{n}\{\text{m}\left(\text{t-h}\right)\}\text{—man}\left\{\text{n}\left(\text{t}\right)\right\}\right]$$

The results are evaluated based on the proximity to the normative data. The cases with different lighting conditions and clothing conditions are compared to see their effect. The efficacy of this markerless system is assessed by comparing it with the Kinect-based system for all conditions.

## Results

### Comparison of OpenPose With Normative Data

**FIGURE 3:**
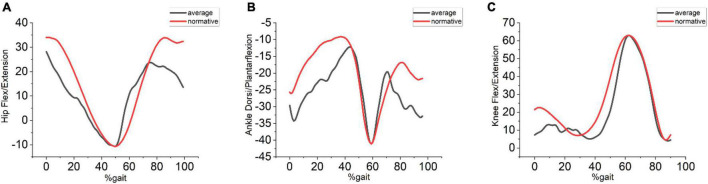
**(A)** Hip flexion/extension **(B)** Ankle dorsi/plantarflexion **(C)** Knee flexion/extension. Average values of all the subjects data using the proposed system shown in blue color and the average values from all the subjects from the gait database shown in red color.

**FIGURE 4:**
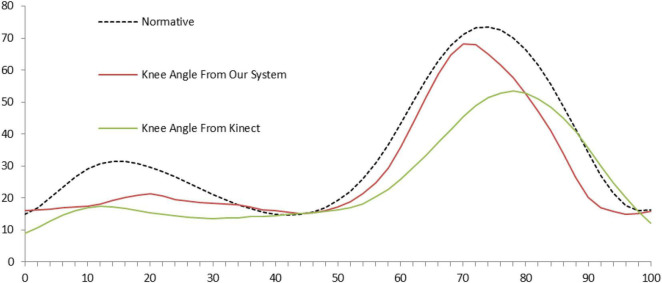
Comparison of knee extension/flexion angle measured using OpenPose and Kinect.

To assess the efficacy of the OMGait the hip, knee, and ankle flexion/extension angles were compared to the values from the normative database (mentioned in the Data Analysis section). For default lighting of 320 lux with the subjects wearing conventional clothes the mean absolute error calculated between OMGait considering all the subjects and the normative database value for hip flexion/extension angle is 7.73°, knee flexion/extension angle is 5.82° and ankle flexion/extension angle is 7.73°. These error values are given in Table 1. Visual comparison can be made between OMGait kinematics and the normative database from Figure 3. Qualitatively we can see that most of the error in the case of hip flexion/extension angle is during the ending phase of the gait whereas, in the case of ankle the beginning and the ending phases reveal large deviation from the normative values. These results clearly show that the OMGait has reasonable accuracy and can be used for the applications discussed earlier. A qualitative comparison between OMGait and the Kinect system from Figure 4 reveals that OMGait performs better.

**TABLE 1:** The error between normative data and the proposed system with default settings.

Joint angle measured	Mean absolute error (°)
Hip	7.73
Knee	5.82
Ankle	7.13

### Further Comparison of Kinect System With OpenPose System

#### Variation in Ambient Lighting

**FIGURE 5:**
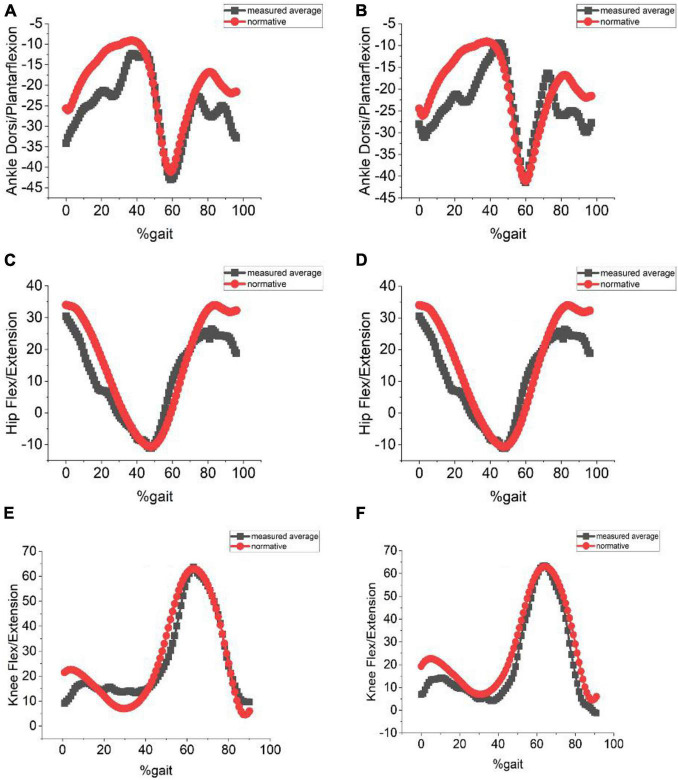
**(A)** Ankle dorsi/plantarflexion measured under bright lighting of 9,800 lux. **(B)** Ankle dorsi/plantarflexion measured with participant in dark lighting of 50 lux. **(C)** Hip flex/extension measured with subject in bright lighting of 9,800 lux. **(D)** Hip flex/extension measured with participant in dark lighting of 50 lux. **(E)** Knee flex/extension measured with participant in bright lighting of 9,800 lux. **(F)** Knee flex/extension measured with participant in dark lighting of 50 lux.

In our previous study using the markerless MS Kinect system, we observed that kinematics of the joint angles differed considerably with lighting conditions, especially in dark and bright conditions. For extremely dark and bright conditions, the pose was not even identified. To study the effect of different lighting conditions on the proposed system, gait measurements under dark (40–100 lux) and very bright (6,000–9,800 lux) lighting conditions were performed. Kinematics measured under these two lighting conditions were compared with the normative dataset. The error values of hip, knee, and ankle angles were less than 9° using OMGait (Table 2). Average values of the hip, knee, and ankle angles measured using OMGait with the normative dataset are shown in Figure 5.

**TABLE 2:** Error in joint kinematic measurement with variation in ambient lighting.

	Dark lighting (50 lux)		Bright lighting (9,800 lux)	
	Mean error	RMSE	Mean error	RMSE
Knee	5.82	6.91	4.28	5.65
Hip	6.72	8.13	5.74	6.90
Ankle	6.07	6.74	5.74	6.72

#### Variation in Subject’s Clothing

The effect of different clothing on the kinematics is measured using the proposed system.

**FIGURE 6:**
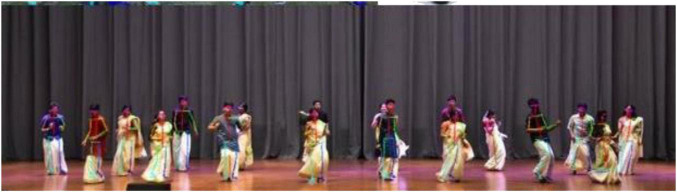
Shows non-conventional clothing namely saree and dhoti.

**FIGURE 7:**
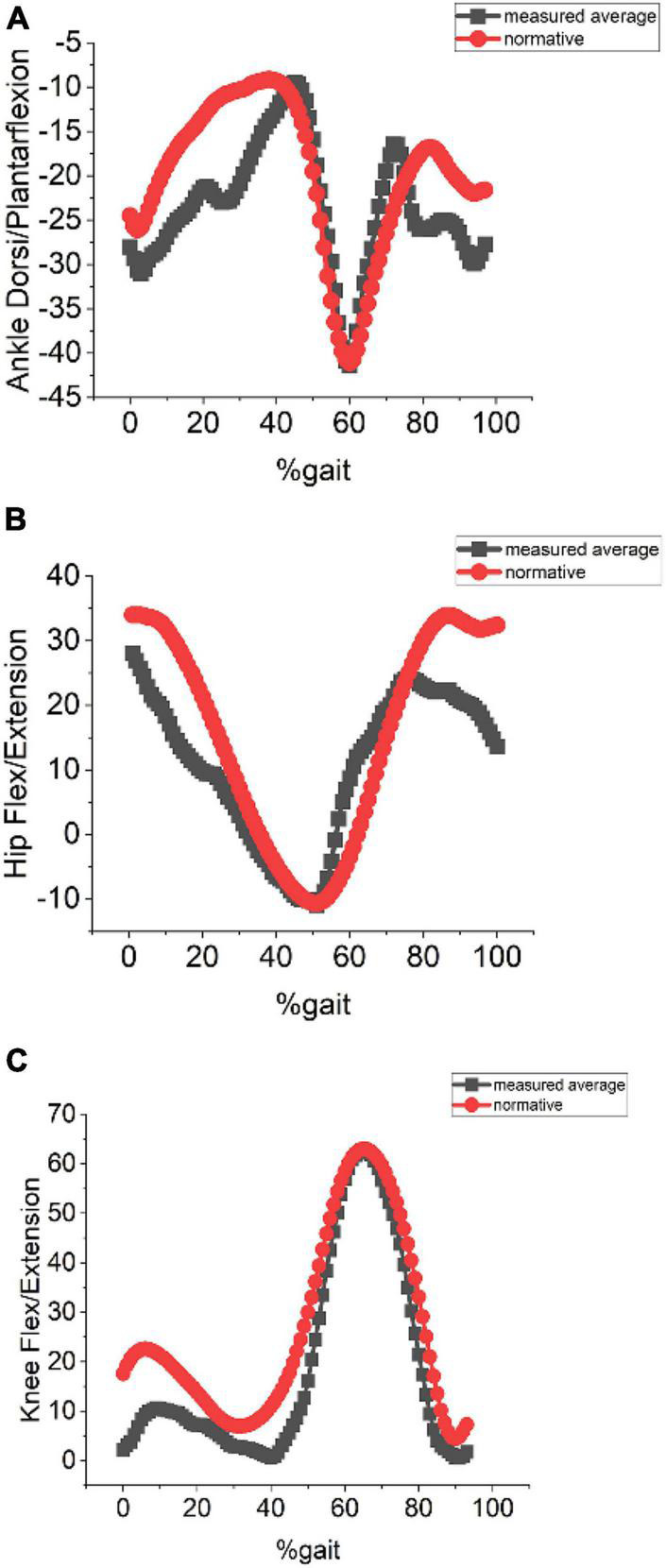
**(A)** Ankle dorsi/plantarflexion. **(B)** Hip flex/extension. **(C)** Knee flex/extension measured with participant wearing non-conventional clothing and under average ambient lighting of 320 lux.

This system was able to detect the pose even when the subjects were wearing non-conventional clothing as shown in Figure 6. The error measured in comparison with the normative data as shown in Table 3 was below 12°. Kinematic values for each of the joints for non-conventional clothing measured using the proposed system were compared with the normative dataset and are shown in Figure 7.

**TABLE 3:** Comparison between Kinect and the proposed system for different dress attire with normative database.

Type of dress worn by the subjects	Joint angle measured	Mean absolute error in angle between Kinect and normative data (in degree)	Mean absolute error in angle between OpenPose and normative data (in degree)
Conventional clothing	Hip	7.35	5.97
Conventional clothing	Knee	7.02	7.26
Conventional clothing	Ankle	Not able to measure	8.10
Non-conventional clothing	Hip	Not able to measure	11.36
Non-conventional clothing	Knee	Not able to measure	4.26
Non-conventional clothing	Ankle	Not able to measure	6.60

## Discussion

This study attempted to build an affordable, non-intrusive markerless gait analysis system (OMGait) capable of measuring gait kinematics irrespective of lighting and clothing conditions. The new gait system consisted of a smartphone and computer to collect kinematics video data, and OpenPose was used to analyze the data. The main findings of this study are (1) hip, knee, and ankle joint kinematics can be measured with relatively good accuracy using a common mobile phone camera and a personal computer using OpenPose. (2) Unlike MS Kinect, kinematic measurements done using OpenPose are tolerant to variations in ambient lighting and the type/kind of dress worn by the subjects.

Recent developments in the field of artificial neural networks (ANN) and computer vision have attracted much attention owing to their ability to solve complex problems with much ease. CNN are explicitly designed for the analysis and study of digital images (Rawat and Wang, 2017). They are employed in many applications like image classification, facial recognition, edge detection, scene labeling, and semantic segmentation (Egmont-Petersen et al., 2002). One of the important applications of CNN is in human pose estimation (Tompson et al., 2014). It refers to the extraction of key anatomical joint coordinates from the digital image of a human body. Our results demonstrate that reasonable accuracy in gait kinematic parameters can be obtained using an approach that combines a simple image/video capturing system (a mobile phone camera) with sophisticated algorithms based on CNN. We believe the CNN played a major role in the OpenPose algorithm in detecting the pose as accurately as possible, which eventually led to a good estimation of the joint angles. In addition to this, the BODY_25 pose model also may have played an important role in detecting more keypoints than MPI and COCO models. The mean error value for all the joint angles is less than 8° using our proposed system. This we believe can further be reduced if (1) the data capturing protocol can be made uniform across all the subjects, (2) Also, capturing more number of walking trails can reduce the variability across each trial and thereby the error values.

**FIGURE 8:**
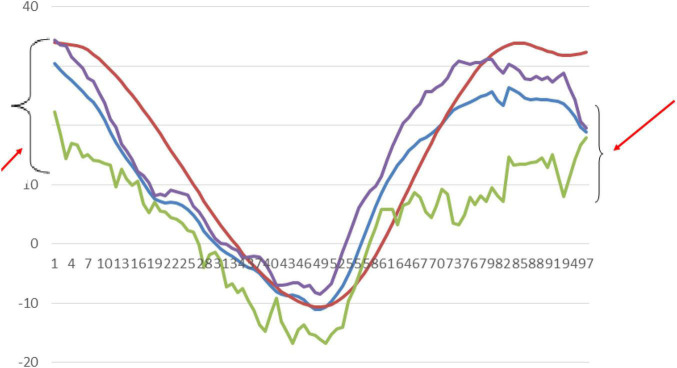
Showing (red arrows) variations at the start and end of gait cycles in our subjects.

In this study, the subjects were not asked to start and stop their gait cycles uniformly, for example by starting with ipsilateral leg push-off. Due to this, when we average the 3 trials obtained from a subject the individual variations in each trial could lead to more error (Figure 8). So, with inter-individual variations in the pace of walking and the gait initiation process, the variability could be more. Despite all these variations, our proposed system shows a mean error value for all the joint angles less than 8 degrees. We propose that this error value will be further reduced if the subjects were asked to walk at a certain pace and were asked to initiate and end their gait cycles uniformly.

Qualitative comparison between OMGait and normative data (Figure 3) shows that OMGait has reasonable accuracy. The error at the start and end of the gait cycles is noticed more in the case of ankle and hip flexion/extension angles. The knee flexion/extension does not show much error with the normative data. The error seen with the hip and ankle angles at the end and beginning of the gait cycle is probably due to inter-individual variations in the gait initiation and termination process.

OMGait performs better than the Kinect system as seen in its comparison with the normative database (Figure 4). OpenPose detects the joint coordinates more accurately when compared to the depth imaging. OMGait uses BODY_25 model which is robust and accurate for different ambient conditions.

In addition, based on our experience, we hypothesize that if the OpenPose algorithm is trained using the gait data obtained from people with different heights and weights, at different gait speeds, different gait initiation and termination steps, clothing, and lighting conditions, the error between OpenPose and normative data will reduce considerably. Also, OpenPose algorithms can accurately detect the pose of the person of interest from multi-person background images/videos and in extremely dark and bright conditions.

During data collection, we observed that when multiple persons were present in the background, OpenPose was able to accurately delineate the pose of all the people in the frame. This feature of OpenPose is helpful for gait measurements of patients in clinical settings without an elaborate setup in the presence of their caregiver or rehabilitation therapist.

**FIGURE 9:**
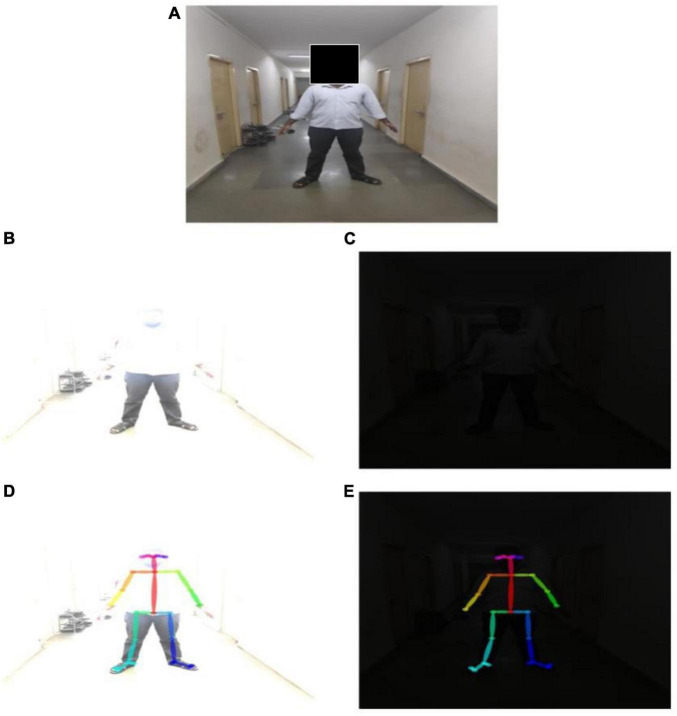
Body pose estimation for extreme lighting conditions. **(A)** Original image. **(B)** Whitewashed image. **(C)** Darkened image. **(D)** BODY_25 pose estimation for whitewashed image. **(E)** BODY_25 pose estimation for darkened image.

OMGait captured the gait parameters fairly accurately in extreme lighting conditions. These results were not surprising since we noticed that the pose estimation was very accurate when applied to post-processed test images. Test data of a darkened (Figure 9C) and a whitewashed image (Figure 9B) are created from one of our datasets (Figure 9A). OpenPose was able to detect the pose for these extreme lighting conditions (Figures 9D,E). This leads us to an important conclusion that the proposed system does not require a dedicated facility to operate.

Our previous work (Parimi et al., 2017) toward developing an affordable gait analysis system using MS Kinect revealed several drawbacks. This system failed to detect the pose in subjects when lighting and clothing conditions were not optimal. The MS Kinect system revealed significant differences in the kinematics of joint angles for conventional and non-conventional clothing. In the case of non-conventional clothing, Kinect even failed to detect the pose. Hence, the joint angles could not be calculated. Even with conventional clothing, the Kinect system was unable to estimate the ankle angle. Furthermore, the Kinect system could not capture more than 1.5 gait cycles due to limitations caused by the depth imaging approach adopted for image processing. The Kinect-based system has shown that the error in skeletal tracking is approximately 10 cm and the accuracy of the depth image produced decreases even beyond 4 cm (Han et al., 2013). Further, markerless systems use multiple cameras to reconstruct the kinematics (Corazza et al., 2006; Mundermann et al., 2006).

The most challenging aspect of any imaging capturing system used for gait analysis is the human pose estimation. To mention some, the technical problems associated with pose estimation are (A) lighting conditions, (B) occlusions from other objects in the image, (C) the inherent high dimension associated with pose estimation, and (D) losing 3D information while measuring pose from 2 D image planes. Our proposed system (OMGait) was able to overcome all the above limitations. The results (section “Further Comparison of Kinect System With OpenPose System”) indicate that the tolerance of OpenPose to extremely bright and dark ambient lighting variations is high (Figure 5). It is observed that pose and keypoint estimation in OpenPose is possible even for extremely whitewashed or darkened images. A potential reason for this superior performance of the proposed system compared to Kinect is the CNN and the BODY_25 model of OpenPose. The CNN in OpenPose was trained *a priori* extensively to estimate key joint anatomical landmarks/coordinates from images of individuals under a wide range of conditions. Compared to MPI and COCO models, BODY_25 performs better by capturing the descriptors for the feet and pelvic center. These descriptors played a significant role in detecting the ankle angles, which was impossible with the Kinect system. The body parts are then assembled using an elegant vector algebra based approach called PAF. The tricky part is assembling the detected parts is to encode not only the position of each limb but also its orientation. Encoding position alone can lead to false associations, as shown in their original work (Cao et al., 2018). The PAF is a 2D vector field that encodes the direction from one part of the limb to the other in each pixel. Each limb has a corresponding PAF associating body parts with high confidence even in a multi-person background image. We believe that PAF has played a significant role in the robust estimation of pose under the challenging circumstances posed by different lighting and clothing conditions.

In comparison to the wearable conventional kinematic measurement systems, OMGait does not have sensor-based limitations. Muro-De-La-Herran et al. (2014) discusses various wearable and non-wearable gait analysis systems in detail. They reported various limitations associated with the wearable technologies such as gyroscopes, accelerometers, and goniometers. Accelerometers lack precision due to increased segment acceleration since active movements of body parts lead to additional movement artifacts signals along with the acceleration signal (Grimaldi and Manto, 2010). Accelerometers also suffer from drift due to the double integration of linear acceleration signal and integration of angular velocity (Dejnabadi et al., 2005; Domínguez et al., 2013). For goniometers along with drift, the calibration of the sensors is not straightforward. Also, a slight hysteresis can still affect the sensor performance (Domínguez et al., 2013).

Few limitations of the proposed system are (a) OMGait shows large deviations at the start and end of the gait cycle and (b) it is not validated by comparing the data from the same subjects with the benchmark systems such as VICON. The error seen with the OMGait and the normative data at the start and end of the gait cycle can be corrected using post processing methods. Validating the proposed system by comparing the data from the same subjects from the VICON system will confirm our findings. The study shows that lighting and clothing conditions do not affect the error in the gait measures for the proposed system. The error seen in the results is predominantly due to the systematic error in the proposed system and not the ambient conditions. We have not attempted minimizing this systematic error. Removing the error from the start and the end of the recording and incorporating the correction due to variation in data collection can achieve this.

Future work should validate our findings by measuring gait parameters hip flexion/extension, ankle dorsiflexion/plantar flexion, and hip flexion/extension using the developed system in clinical conditions such as stroke or spinal cord injury patients. Moreover, the proposed system can be used as the backend for an android application that will measure gait metrics in real time. This study is the first step in developing such a system. Future work of this system should include development of an android app as it is fairly accurate (compared to Kinect), computationally inexpensive, and works in any background, lighting, and clothing conditions.

## Conclusion

We have introduced here a computer vision based approach to human gait analysis. We propose a robust, cost-effective, markerless, and user-friendly gait analysis system using a computer and a mobile phone camera. Our proposed system certainly overcomes the drawbacks of conventional kinematics measurement systems like inertial sensors, goniometers, accelerometers. Also, unlike the external marker based systems such as VICON, it does not require a dedicated gait analysis lab facility or the external fiducial marker placement. It also overcomes the limitations of markerless systems such as Kinect that is prone to large variations in ambient lighting and clothing. OpenPose algorithm used is a free and open-source pose estimation library. This system can be further developed to be more robust and accurate by decreasing errors through post-processing and standardizing the gait measurement protocol.

While OpenPose was used previously in pose estimation, its application in gait measurement systems is limited. Capturing gait in extreme light conditions (bright and dark) has not been studied before. Clothing that is not conducive to gait analysis is also a challenge that this study highlights. The studies we have seen with smartphone cameras for gait analysis use multiple cameras or moving camera systems for data capture, whereas ours is static hence easier to use. All these observations make our study feasible, accessible and novel.

## Data Availability Statement

The datasets presented in this article are not readily available because it is proprietary property of the parent institute. Requests to access the datasets should be directed to the corresponding author.

## Ethics Statement

Ethical review and approval was not required for the study on human participants in accordance with the local legislation and institutional requirements. The patients/participants provided their written informed consent to participate in this study.

## Author Contributions

AV conceived the idea, collected data, processed the data, and wrote portions of the manuscript. VR collected the data, analyzed the results, and wrote the manuscript. TR analyzed the results and wrote the manuscript. PG provided consultation on the data analysis and interpretation, made extensive revision on the manuscript. CP collected the data, processed the data, analyzed the results, wrote the manuscript, and overall supervision of this study. All authors contributed to the article and approved the submitted version.

## Conflict of Interest

The authors declare that the research was conducted in the absence of any commercial or financial relationships that could be construed as a potential conflict of interest.

## Publisher’s Note

All claims expressed in this article are solely those of the authors and do not necessarily represent those of their affiliated organizations, or those of the publisher, the editors and the reviewers. Any product that may be evaluated in this article, or claim that may be made by its manufacturer, is not guaranteed or endorsed by the publisher.
